# The Influence of Cold Therapy on the Physical Working Capacity at the Electromyographic Threshold for Consecutive Exercise Sessions

**DOI:** 10.3390/bioengineering11030292

**Published:** 2024-03-21

**Authors:** Rami E. Maasri, Jonathan R. Jarvie, Jacob S. Karski, Logan J. Smith, Moh H. Malek

**Affiliations:** 1 Physical Therapy Program, Department of Health Care Sciences, College of Pharmacy and Health Sciences, Wayne State University, Detroit, MI 48201, USA; 2 Integrative Physiology of Exercise Laboratory, Department of Health Care Sciences, College of Pharmacy and Health Sciences, Wayne State University, Detroit, MI 48201, USA; 3 Eugene Applebaum College of Pharmacy & Health Sciences, Wayne State University, 259 Mack Avenue, Room 2248, Detroit, MI 48201, USA

**Keywords:** exercise physiology, motor unit recruitment, neuromuscular fatigue

## Abstract

Background: The purpose of this study was to determine whether cold therapy after the first exercise test influences the physical working capacity at the fatigue threshold (PWC_FT_) during the second exercise test. We hypothesized that cold therapy would delay the onset of PWC_FT_ for the second exercise test relative to the control visit (i.e., no cold therapy). Methods: Eight healthy college-aged men volunteered for the present study. For each of the two visits, subjects performed incremental, single-leg, knee-extensor ergometer, followed by either resting for 30 min (control visit) or having a cold pack applied for 15 min and then resting for 15 min (experimental visit). Then, the same exercise test was performed. The order of visits (control vs. experimental) was randomized for each subject. The exercise indices and PWC_FT_ were determined for each of the two visits and statistically analyzed using two-way repeated measures analysis of variance. Results: The results indicate no significant (*p* > 0.05) mean differences for maximal power output, heart rate at end-exercise, and PWC_FT_ between the control and cold therapy visits. Moreover, there were no significant (*p* > 0.05) mean differences between the first and second exercise workbout within each visit. Conclusions: The findings of this study suggest that cold therapy did not influence neuromuscular fatigue.

## 1. Introduction

Recovery from any activity, such as exercising in the gym or completing an athletic event (i.e, marathon), is a critical component of reducing potential injury to the muscles [1,2]. These modalities use cold-water immersion or whole-body cryotherapy target muscles and joints so that there is an accelerated return of physiological parameters to basal levels from acute inflammation or local swelling [3]. Studies determining the efficacy of cooling the body on human performance have primarily used cold-water immersion [4,5,6] or cold packs [7,8]. While the former is typically used by athletes, the latter is often a more convenient and economical approach that anyone with access to ice cubes and a zip-lock bag can apply to a body part.

To allow for more inclusivity of studies that used various deliveries of cold treatments, the term ‘cold therapy’ throughout the manuscript will encompass the use of cold-water immersion or cold packs. The effect of cold therapy on delaying neuromuscular fatigue has become an area of interest in recent years but studies are equivocal [4,9,10]. Loro et al. [11] reported a significant increase (~38%) in surface electromyographic (EMG) activity in the quadriceps femoris muscles following cold therapy. It is hypothesized that cold therapy may potentially decrease muscle inhibition [11,12,13,14]. Yona [15] reported that the cooling the skin at the surface increases motor unit recruitment. Indeed, the use of EMG provides a practical, non-invasive methodology that could determine the motor unit recruitment and firing rate of the working muscle for a given exercise modality and intensity [16]. Macedo and colleagues [4] reported that cold therapy differentially influences the muscle recruitment patterns of the lower limb during vertical-jump-landing between collegiate basketball players and healthy college-aged men. Specifically, the authors found that cold therapy decreased the electromyographic activity of the lower limb [4]. Wakabayashi et al. [10] found that maximal isometric knee extension was significantly lower and corresponded to a lower EMG response following cold therapy relative to the control condition. Conversely, Delahunty and colleagues [17] reported that cold therapy increased the EMG amplitude of the treated limb and reduced the inhibitory activity of motor networks in both hemispheres, which, in turn, increased motor network activity in both hemispheres. To our knowledge, no studies have examined the influence of cold therapy on neuromuscular fatigue for continuous muscle actions, such as those associated with treadmill running or cycle ergometry.

Originally developed by deVries and colleagues in the 1980s, the physical working capacity at the fatigue threshold (PWC_FT_) [16] is an approach to measure the change in the EMG amplitude versus time relationship for incremental cycle ergometry [16]. The PWC_FT_ is the highest power output (i.e., exercise intensity) that an individual can maintain indefinitely without an increase in the EMG amplitude. In brief, the EMG amplitude versus time relationship is analyzed using linear regression for each incremental stage. This is derived from calculating the average value between the highest power output with a nonsignificant (*p* > 0.05) slope and the lowest power output with a significant (*p* < 0.05) positive slope [16].

Studies have validated the PWC_FT_ [18], as well as showing it to be influenced by different interventions [16]. To date, however, no studies have examined the effect of cold therapy on the PWC_FT_. The purpose of this study, therefore, was to determine whether cold therapy after the first exercise test influences the PWC_FT_ during the second exercise test. We hypothesized that cold therapy would delay the onset of PWC_FT_ for the second exercise test relative to the control condition (i.e., no cold therapy).

## 2. Materials and Methods

### 2.1. Overall Research Design

**Figure 1:**
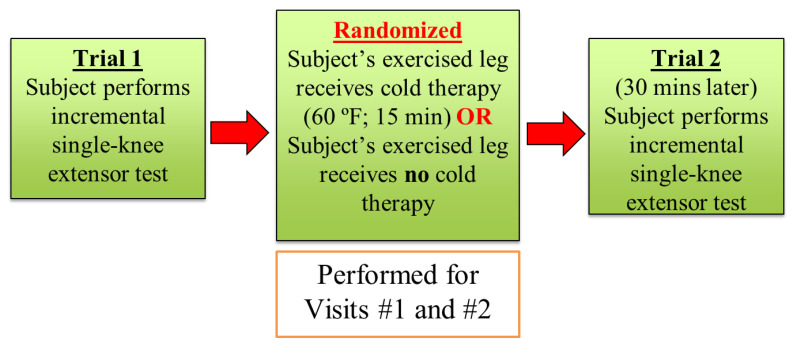
Overall experimental design of the present study.

An overall depiction of the cross-over randomized control design is shown in Figure 1. In brief, subjects visited the laboratory on 2 occasions separated by 7 days. For each visit, subjects had EMG electrodes placed on their rectus femoris muscle of the non-dominant leg (based on kicking preference). Overall, subjects performed an incremental single-leg knee-extensor ergometer test (i.e., Trial 1) then rested for 30 min, followed by their performing the same incremental single-leg knee-extensor ergometer test a second time (i.e., Trial 2). Specifically, for the cold therapy visit, each subject would have a cold pack placed on the rectus femoris muscle for 15 min after the first exercise test. After the cold therapy, subjects rested for 15 min and then performed the second single-leg knee-extensor ergometer test. For the control visit, the same sequence of events was performed, but the subject rested for 30 min with no cold therapy intervention.

### 2.2. Subjects

The number of subjects was determined by performing a power analysis based on previous studies (effect size = 1.41; β = 80%; and *p* < 0.05) [19,20,21,22]. Therefore, eight healthy college-aged men [mean ± SEM: age, 24.3 ± 0.4 y; weight, 88.1 ± 4.3 kg; and height, 1.83 ± 0.04 m] ranging from 23 to 26 years old volunteered for the present study. All subjects were instructed to avoid exercising their legs the day prior to the testing sessions and avoid the consumption of caffeine on the testing days [19,20,21,22]. In addition, the subjects were tested at the same time (±1 h) for both experimental and control visits. All procedures were approved by the University Institutional Review Board for Human Subjects, and each subject signed an informed consent prior to testing.

### 2.3. Incremental Single-Leg Knee-Extensor Ergometry

The single-leg knee-extensor ergometer test was used, which allows for subjects to exercise the quadriceps femoris muscles with minimal engagement of other muscles, such as the hamstrings [19,20,21,22]. Importantly, Richardson et al. [23] showed that the rectus femoris muscle is the primary muscle involved in this exercise, followed by the vastus lateralis, vastus medialis, and vastus intermedius. Moreover, the main goal of this exercise modality is to limit the contribution of cardiovascular and ventilatory responses, which are usually seen in whole-body exercise such as cycle ergometry and treadmill running [19,20,21,22]. In brief, the subject is seated in a semirecumbent position, and the non-dominant leg (based on kicking preference) is placed in a boot, which is connected via a metal rod to the crank handle of a cycle ergometer [19,20,21,22]. The motion of the leg follows an arc trajectory starting at ~90° and ending at ~160–170° [19,20,21,22]. Therefore, the subject never reaches maximal extension of the exercise leg. The dominant leg rests on a platform during the exercise test.

The incremental protocol consists of starting at 4 W for 2 min, followed by an increase of 4 W every minute until the subject is unable to maintain the 70 revolutions per minute (rpm) kicking cadence [19,20,21,22]. In addition, the Modified Borg Rating of Perceived Exertion (0–10) was recorded at the end of each workload. Moreover, heart rate throughout the exercise test was documented using a Polar Heart Rate Monitor (Polar T31C; Polar Electro Inc., Kempele, Finland) [19,20,21,22].

### 2.4. Cold Therapy Protocol

During the cold therapy visit, each subject had a large cold pack placed on the quadricep femoris muscles with a specific emphasis on cooling the rectus femoris muscle. The use of a cold pack as opposed to cold-water immersion is more akin to the treatment modality physical therapists use in rehabilitation settings. Once the target skin temperature of 15 °C was reached, the 15 min of cold therapy began. Skin temperature was recorded using a thermometer (Model SDL200; Extech 4-Channel, Nashua, NH, USA), which was taped to the skin under the cold packs. Approximately 4–5 min was needed for each subject to reduce the skin temperature to the target temperature (i.e., 15 °C). For each visit (i.e., control and cold therapy), surface skin temperature for the rectus femoris was recorded on four occasions: (1) prior to the start of exercise for Trial 1; (2) prior to the start of exercise for Trial 2; (3) immediately at end-exercise of Trial 1; and (4) immediately at end-exercise of Trial 2.

### 2.5. Electrode Placements

The rectus femoris was used to record the EMG signal, because this is the primary muscle for this mode of exercise. In brief, bipolar (20 mm center-to-center) surface electrodes (EL500-6, BIOPAC Systems, Inc., Santa Barbara, CA, USA) were placed over the muscle belly, corresponding to 50% of the distance between the anterior superior iliac spine and superior border of the patella [19,20,21,22]. The reference electrode was placed over the iliac crest. The shaved skin at each electrode site was carefully abraded and cleaned with alcohol, and interelectrode impedance was kept below 2000 ohms, consistent with our previous work [19,20,21,22]. The EMG signal from each electrode placement site was amplified (gain: ×1000) using differential amplifiers (EMG 100B, BIOPAC Systems, Inc., Santa Barbara, CA, USA). A permanent marker was used to trace the outline of the electrode placement for consistency in subsequent visits.

### 2.6. Determining the PWC_FT_

**Figure 2:**
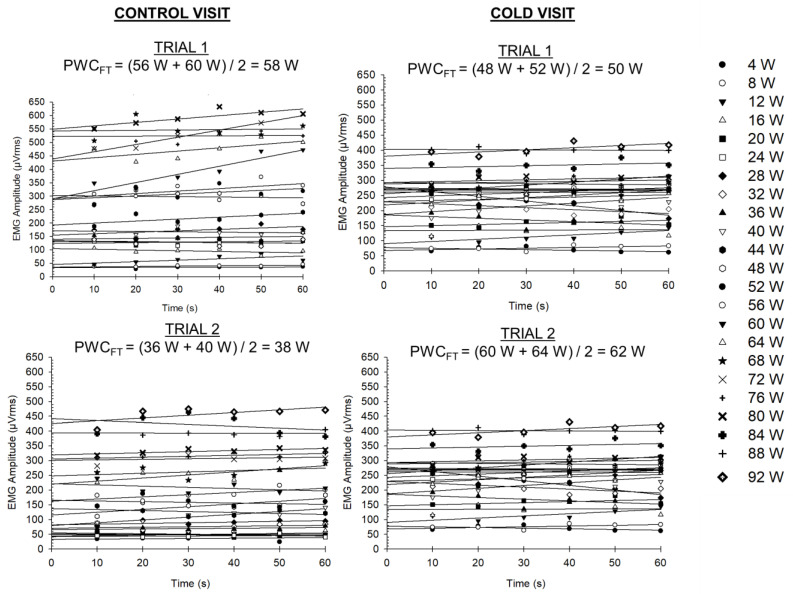
Representative time vs. absolute EMG amplitude plots for each power output for all four experimental conditions for a single subject.

As shown in Figure 2, for each stage, the EMG amplitude (microvolts root mean square (µVrms)) was calculated from 10 s epochs for each stage. The power output for each stage was increased every minute. This modified protocol is a departure from the traditional two-minute stage that is used, because Khan and colleagues [24] reported that the PWC_FT_ derived from the one-minute stage was not statistically different than the PWC_FT_ derived from the two-minute stage. Nevertheless, for each stage, a linear regression was performed for the EMG amplitude versus time relationship to identify the slope of the regression line. Thereafter, the highest power output with a non-significant (*p* > 0.05) slope coefficient and the lowest power output with a significant (*p* < 0.05) positive slope coefficient were identified. The average of these two power outputs was then calculated to determine the PWC_FT_. These steps were performed for each trial, leading to four PWC_FT_ values (see Figure 2).

### 2.7. Recording the EMG Signal and Processing

Using a laptop (Dell Inspiron E1705, Dell Inc., Round Rock, TX, USA), the EMG signal was digitized at 1000 Hz and then subsequently processed using a custom program (LabVIEW; version 2019, National Instruments, Austin, TX, USA). A fourth-order Butterworth bandpass filter at 10–500 Hz was used, and the EMG amplitude for each subject and visit was calculated as the average of all the completed bursts during a 10 s epoch [19,20,21,22].

### 2.8. Statistical Analysis

All data presented in the present study are mean ± standard error of the mean (SEM) values. Separate 2 [Trial: 1 and 2] X 2 [Experimental Visit: control or cold therapy] repeated measures Analysis of Variance (ANOVAs) were used for variables such as maximal power output, absolute and normalized PWC_FT_, absolute and normalized (%max) heart rate at end-exercise, and RPE at end-exercise. In addition, the slopes of the regression lines for the power output as a percentage of maximal completed power output versus RPE relationship were statistically compared to identify potential differences in leg fatigue perceptions between trials. All statistical analyses were performed using Prism 8 (GraphPad Software; San Diego, CA, USA) with an alpha level set at *p* ≤ 0.05.

## 3. Results

### 3.1. Incremental Test and PWC_FT_

As shown in Table 1, the mixed factorial ANOVA revealed no significant group X experimental visit interaction [F(1,14) = 0.00; *p* = 0.999] for maximal power output. In addition, there were no significant main effects for group [F(1,14) = 0.173; *p* < 0.684]. Furthermore, there were no significant main effects for experimental visit [F(1,14) = 3.818; *p* = 0.071].

The mixed factorial ANOVA revealed no significant group X experimental visit interaction [F(1,14) = 0.034; *p* < 0.857] for PWC_FT_. In addition, there were no significant main effects for group [F(1,14) = 0.011; *p* = 0.918]. Furthermore, there were no significant main effects for experimental visit [F(1,14) = 0.304; *p* = 0.590].

The mixed factorial ANOVA revealed no significant group X experimental visit interaction [F(1,14) = 0.024; *p* = 0.879] for relative percent of maximal power output (PWC_FT_). In addition, there were no significant main effects for group [F(1,14) = 1.192; *p* = 0.293]. Furthermore, there were no significant main effects for experimental visit [F(1,14) = 0.830; *p* < 0.378].

The mixed factorial ANOVA revealed no significant group X experimental visit interaction [F(1,14) = 0.00; *p* < 0.992] for heart rate at end-exercise. In addition, there were no significant main effects for group [F(1,14) = 0.027; *p* = 0.872]. Furthermore, there were no significant main effects for experimental visit [F(1,14) = 0.009; *p* = 0.927].

The mixed factorial ANOVA revealed no significant group X experimental visit interaction [F(1,14) = 0.000; *p* < 0.929] for heart rate at end-exercise (percentage of predicted age). In addition, there were no significant main effects for group [F(1,14) = 0.028; *p* = 0.868]. Furthermore, there were no significant main effects for experimental visit [F(1,14) = 0.008; *p* = 0.929].

The mixed factorial ANOVA revealed a significant group X experimental visit interaction [F(1,14) = 9.00; *p* = 0.010] for RPE at end-exercise. In addition, there were no significant main effects for group [F(1,14) = 0.000; *p* = 0.999]. Furthermore, there were no significant main effects for experimental visit [F(1,14) = 1.00; *p* = 0.334]. The post-hoc Tukey honest significance test (*HSD*) revealed that the RPE at end-exercise between trial 1 and trial 2 for the control visit was statistically significant.

### 3.2. Comparison of Surface Skin Temperature

The mixed factorial ANOVA revealed a significant group X experimental visit interaction [F(1,14) = 88.127; *p* < 0.001] for surface muscle temperature (Table 2). We also found significant main effects for group [F(1,14) = 28.40; *p* < 0.001] and experimental visit [F(1,14) = 4.679; *p* = 0.048]; however, these results were not further analyzed due to the significant interaction [25]. The post-hoc Tukey *HSD* test revealed significant mean differences between trials 1 and 2 within each experimental visit. Moreover, we found that surface muscle temperature prior to starting the second exercise test was significantly lower after cold therapy relative to the control visit (Table 2).

### 3.3. Comparison of Slopes for RPE Index

**Figure 3:**
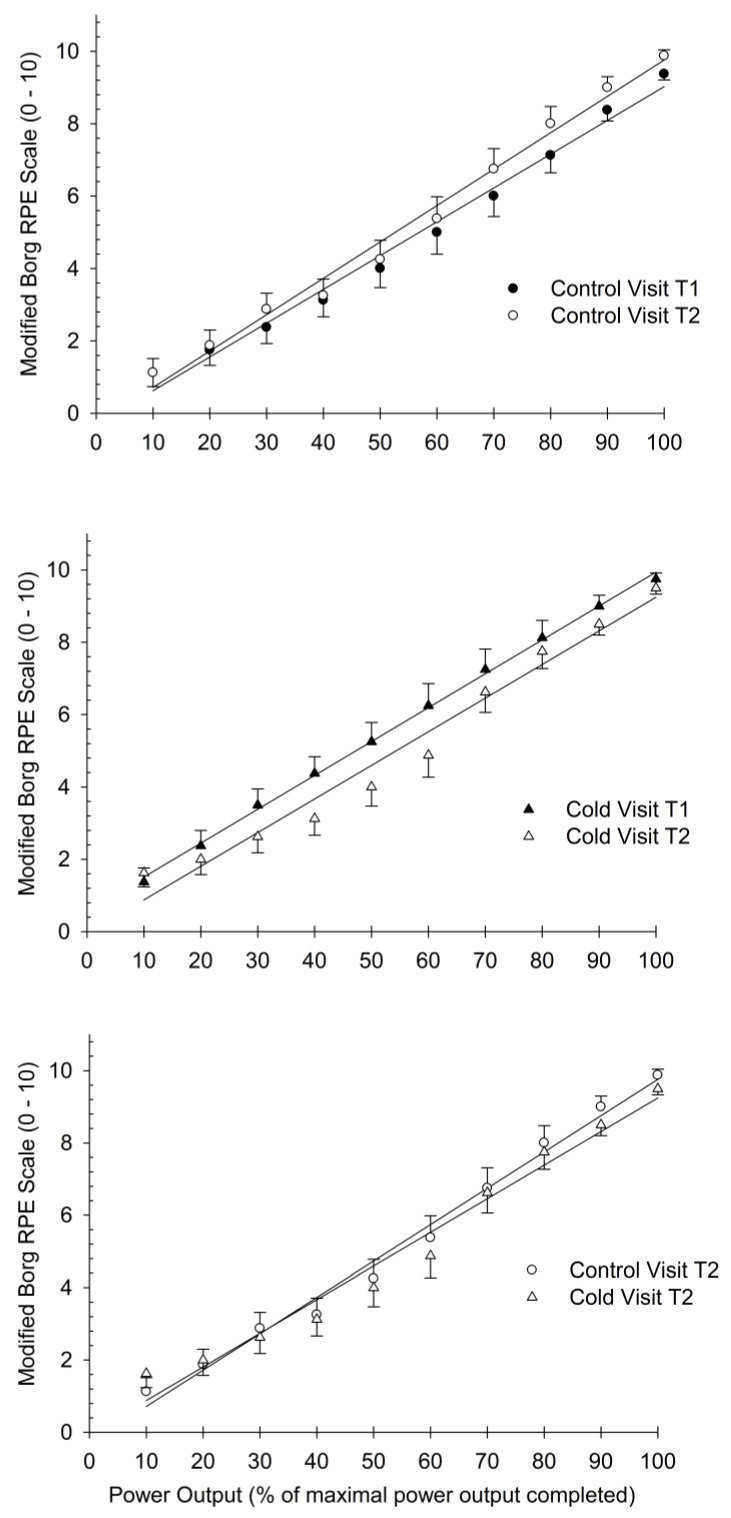
Comparison of slopes between trial 1 and trial 2 for the control (**top panel**) and cold therapy (**middle panel**) visits. We also compared slopes between the control and cold visits for trials 2 (**bottom panel**). For all three graphs, there were no statistically significant differences between the slopes of the regression lines. Data are presented at mean ± SEM.

As shown in Figure 3, we compared the slopes of the regression line for the power output versus RPE relationship between trials 1 and 2 for the control (top panel) and cold (middle panel) visits. In addition, the slopes of the regression line for the power output versus RPE relationship between trials 2 for the control and cold visits (bottom panel) were also compared. In all instances, there were no significant differences between the slopes for the three plots (Figure 3).

## 4. Discussion

The main finding of the present investigation was that 15 min of cold therapy following incremental, single-leg, knee-extensor ergometer to voluntary exhaustion did not enhance exercise performance for the same test when performed a second time. We did find, however, that surface muscle temperature measured prior to the start of the exercise test was significantly different for trial 2 relative to trial 1 within the two experimental visits (Table 2). Moreover, we found that the end-exercise surface muscle temperature for trial 2 in the cold therapy visit was significantly lower than the surface muscle temperature for trail 2 in the control visit. Nevertheless, these changes in skin temperature did not delay the onset of the PWC_FT_.

### 4.1. Use of Cold Therapy

One of the main uses of cold therapy after an exercise session is to reduce the perception of discomfort or potential fatigue [26,27,28,29,30,31]. This may be especially true for sporting competitions where the athlete is competing in multiple events within the same day (i.e., track and field or swimming) with limited time to rest between events. Moreover, it is customary practice in training rooms, as well as rehabilitation clinics, to use cold therapy for the treatment of musculoskeletal injuries [26,27,28,29,30,31]. Indeed, the utility of cold therapy resides in its reducing skin and muscle temperature, which then reduces blood flow and the metabolism of the target muscle [26,27,28,29,30,31]. As a result, physiological responses such as edema, muscle spasm, or inflammation are reduced [26,27,28,29,30,31]. Pournot and colleagues [31] reported that cold therapy significantly reduced the inflammatory response following 45 min of high-intensity running when compared to the control visit (i.e., no cold therapy). In a meta-analytic review, Bleakley and colleagues [32] examined the utility of cold therapy on delayed onset muscle soreness. The investigators reported that cold therapy significantly reduced levels of pain for 24 h after the intervention, with effects lasting up to 96 h [32]. Liao and Xu [30] reported, in a group of patients undergoing total knee arthroplasty, that cold therapy improved postoperative swelling and reduced the amount of time patients remained in bed. This improved the patient’s self-care and quality of life postoperatively [30].

### 4.2. Utility of the Single-Leg Knee-Extensor Ergometer

In the present study, the exercise modality we used isolates the quadriceps femoris muscles [16]. Moreover, studies indicate that the oxygen consumption of the working muscles (i.e., quadriceps femoris) at maximal exercise for the single-leg knee-extensor ergometer are approximately two to three times higher than the oxygen consumption for the same muscles during cycle ergometry [33,34,35]. For example, Ru and Hallén [36] reported that mass-specific oxygen uptake (i.e., dividing the net oxygen uptake by the active muscle mass during exercise) was significantly higher for single-leg knee-extensor ergometer versus cycle ergometry (~0.400 vs. ~0.015 L of oxygen per kg of muscle mass per min). In addition, studies have compared the EMG amplitude for the quadriceps femoris muscles, using incremental testing, between the two exercise modalities (single-leg cycle ergometry and single-leg knee-extensor ergometer) and found that the EMG amplitude is ~40% higher at maximal exercise for single-leg knee-extensor ergometer versus single-leg cycle ergometry [37]. Therefore, in the present study, the incremental single-leg knee-extensor ergometer provided a significant metabolic stress to the quadricep femoris muscles.

### 4.3. Neuromuscular Response and Cold Therapy

The findings of the current research indicated that cold therapy did not significantly change the PWC_FT_ from the first to the second trial (Table 1). An interesting finding, however, is that, for the control visit, we did not see a significant increase in PWC_FT_ for the second trial relative to the first trial. This is contrary to the findings of Bremer and colleagues [38], who reported that a second workbout of incremental single-leg knee-extensor ergometer significantly increased PWC_FT_ by ~26%. One caveat, however, is that subjects in the Bremer et al. [38] study rested for 60 min between trials 1 and 2, whereas in the current study, subjects rested 30 min between trials 1 and 2. Thus, the potential neuromuscular priming of the working muscles from a previous exercise test may require 60 min of rest to be effective on a subsequent exercise test.

Studies have also suggested that cold therapy may hinder motor unit recruitment for a subsequent workout [9]. Indeed, Rupp et al. [39] reported that 15 min of cold therapy did not significantly change performance in the yo-yo intermittent recovery test in a group of collegiate men and women soccer players. Sanchez-Urena and colleagues [9] examined the efficacy of continuous and intermittent cold therapy protocols on neuromuscular fatigue using tensiomyography, which assesses the contractile properties of a target muscle [9]. The authors [9] reported that neither the intermittent nor the continuous cold therapy modalities that were used significantly changed the contractile properties of muscles in the lower extremities. Peiffer et al. [40] reported that cold therapy following exercise in the heat reduced maximal voluntary isometric contraction (MVIC) and MVIC with superimposed electrical stimulation by ~12% and ~14% at 45 and 90 min, respectively, relative to the control visit. Macedo and colleagues [4] reported that cold therapy reduced the EMG amplitude of the lower limb muscles when athletes performed single-leg jump landings. The authors suggested that reduced muscle temperature may potentially interfere with motor unit recruitment during an explosive activity such as jumping [4]. Wakabayashi and colleagues [10] found that EMG mean power frequency (MPF) values, at various intensities of submaximal isometric knee extensions for the rectus femoris and vastus lateralis muscles, were significantly lower in the cold therapy conditions relative to the control condition. The authors concluded that the reduction in EMG MPF may be due, in part, to the decrease in nerve and muscle fiber conduction velocity post cold therapy [10].

### 4.4. Study Limitations

The current investigation may have a few potential limitations that are worth noting. First, our sample size of eight subjects may be considered small, and thus may not capture the effect of our intervention, if one truly exists. Our sample size was based, in part, on intervention studies using PWC_FT_ as the main outcome variable. Moreover, we used a within-subjects design rather than using a between-subjects design, which would potentially result in large variability for the physiological outcomes. Second, we did not use cold-water immersion to cool the muscle; rather, we used cold packs on the surface of the thigh. This decision was, in part, due to logical considerations, as we did not want the EMG electrodes to get wet or to remove them prior to the cold therapy and then replace them with a new set of electrodes. Therefore, total immersion of the thigh may have potentially resulted in a different outcome than our current finding. Third, we did not include women in our study because recent studies recommend that the menstrual cycle needs to be accounted for, since hormonal levels may influence physiological measures [41,42]. Moreover, Nuzzo and Deaner [43] reported that there may be differences between men and women in their willingness to participate in exercise research studies, where men are interested in strength and endurance experiments, whereas women are interested in participating in stretching and group aerobic interventions. Fourth, we did observe a statistically significant mean difference in the RPE value at end-exercise between trials 1 and 2 in the control visit (Table 1). This finding may be due, in part, to the small variation between subjects regarding their RPE value at end-exercise rather than a meaningful physiological difference. That is, the mean values of 9 (trial 1) and 10 (trial 2) for the control visit both indicate the subject’s perceived maximal exertion for the exercised leg. This information, in conjunction with the slope comparison data for RPE as a percentage of the maximal power output that was completed (Figure 3), suggest that RPE may not be an effective measure when subjectively examining perceived exertion associated with cold therapy. Lastly, we used the single-leg knee-extensor ergometer, which is a single-joint exercise used to isolate the thigh muscles and minimize the cardiovascular or ventilatory limitations seen in multi-joint exercises such as treadmill running [22,23,44]. Therefore, future studies may be needed to determine the effect of cold therapy on neuromuscular fatigue using multi-joint exercise.

## 5. Conclusions

In summary, the results of this study indicated that cold therapy did not enhance or reduce the neuromuscular response to voluntary fatiguing exercise. Our study, however, used a unique endurance test that isolated the quadriceps femoris muscles using a single-joint movement. Therefore, future studies may be needed to determine if cold therapy influences neuromuscular fatigue for whole-body exercise such as treadmill exercise. Nevertheless, cold therapy has benefits, such as reducing the inflammatory process and therefore decreasing swelling (or edema) in the tissue of interest. Coaches and athletes should consider the potential impact of cold therapy on subsequent exercise workbouts, particularly when events are scheduled on the same day. Although the present study did not find any significant effects on neuromuscular fatigue, there is an increasing body of literature suggesting that cold therapy may reduce the neuromuscular response to exercise [4,10,39,40]. 

## Figures and Tables

**Table 1:** Outcome variables (mean ± SEM) for the different physiological parameters that were measured.

	Control Visit		Cold Therapy Visit	
Physiological Outcomes	Trial 1	Trial 2	Trial 1	Trial 2
Maximal power output (W)	60 ± 7	63 ± 7	64 ± 7	67 ± 7
PWC_FT_ (W)	33 ± 4	33 ± 4	34 ± 4	32 ± 4
PWC_FT_ (% maximal PO)	58 ± 5	54 ± 5	53 ± 5	48 ± 5
Heart rate at end-exercise (bpm)	144 ± 12	144 ± 14	147 ± 12	147 ± 14
Heart rate at end-exercise (% predicted)	73 ± 6	73 ± 7	75 ± 6	75 ± 7
Modified RPE at end-exercise (0–10 scale)	9 ± 0	10 ± 0 *	10 ± 0	10 ± 0

Note: Trials 1 and 2 were separated by 30 min of rest for each visit. % predicted: 220—age. * Significant difference. (*p* = 0.013) between trial 1 and trial 2.

**Table 2:** Surface muscle temperature (mean ± SEM) for the rectus femoris muscle.

	Control Visit		Cold Therapy Visit	
Surface Muscle Temperature (°C)	Trial 1	Trial 2	Trial 1	Trial 2
Prior to start of the exercise	30.3 ± 0.4	32.5 ± 0.6 *	29.5 ± 0.4 *	26.1 ± 0.6 *†
Immediately at end-exercise	32.3 ± 0.5	32.6 ± 0.7	31.8 ± 0.5	30.5 ± 0.7 **

Note: * indicates significant (*p* < 0.001) mean difference within visit between trials 1 and 2. † indicates significant (*p* < 0.001) mean difference between trials 2 for the control and cold therapy visits. ** indicates significant (*p* = 0.02) mean difference between trials 1 and 2 for the cold therapy visit.

## Data Availability

All data from this study are available from the authors upon reasonable request.
